# Skin AllergoOncology: A Framework for Decoding the Context-Dependent Functions of the IgE–FcεRI Axis in Cutaneous Malignancies

**DOI:** 10.3390/cancers18172807

**Published:** 2026-08-29

**Authors:** Zhengkui Zhang, Bing Wen, Kun Xie

**Affiliations:** Department of Plastic Surgery and Burns, Peking University First Hospital, No. 8 Xishiku Street, Xicheng District, Beijing 100034, China; 08245@pkufh.com (Z.Z.);

**Keywords:** IgE, FcεRI, skin AllergoOncology, cutaneous malignancy, atopic dermatitis, mast cell, tumor immunosurveillance, engineered IgE antibody

## Abstract

The immunoglobulin E (IgE)–FcεRI axis, a central mediator of allergic inflammation, plays a dual role in cutaneous tumor immunity. Epidemiological evidence presents a paradox: atopic dermatitis (AD) promotes keratinocyte carcinogenesis, yet systemic allergic responses are associated with reduced melanoma risk. This review proposes the “Skin AllergoOncology” framework to resolve this paradox. The framework defines the skin as the only human organ in which two antagonistic IgE–FcεRI programs, protective immune surveillance and chronic pro-tumor inflammation, can be spatially juxtaposed. It is organized around three analytical dimensions: IgE repertoire quality, inflammatory kinetics, and effector-cell polarization state. We critically examine the epidemiological AD–keratinocyte carcinoma association, highlighting the confounding role of immunosuppressive therapies, and review the molecular infrastructure enabling dual functional outputs, including antigen focusing by trimeric FcεRI on Langerhans cells and microenvironment-dependent mast cell polarization. We contend that the absence of direct functional evidence for protective IgE in humans represents the central question for the next decade, and we propose a five-year dual-engine roadmap to identify the human protective IgE signature. This framework informs risk-stratified surveillance, engineered IgE antibody therapy, and the long-term oncological safety assessment of anti-allergic biologics.

## Simple Summary

Allergies and cancer may seem like opposite problems, but both involve the immune system. This review focuses on the skin—the body’s largest organ and the place where allergic reactions often first appear. We ask why some allergic conditions, like eczema, are linked to a higher risk of skin cancer, while other allergies seem to be linked to a lower risk of melanoma. Our “Skin AllergoOncology” framework helps explain this puzzle by showing that the same immune pathway can either protect against or promote cancer depending on the circumstances. We propose a five-year research plan to identify the protective immune signature in humans, which could lead to better ways to predict skin cancer risk and develop new treatments.

## 1. Introduction

If systemic allergic reactivity can enhance immune surveillance against melanoma, why does the most intense form of local cutaneous allergic inflammation—atopic dermatitis (AD)—correlate with an increased risk of keratinocyte carcinogenesis? This paradox, which has divided the epidemiological and immunological literature for over a decade, suggests that the immunoglobulin E (IgE)–Fc fragment of immunoglobulin E, high affinity receptor I (FcεRI) axis is not a monolithic signaling module but a context-dependent functional switch whose output is profoundly shaped by the tissue microenvironment [1,2].

This paradox can be resolved by recognizing a unique anatomical feature: the skin is the only human organ in which the two antagonistic functional programs of the IgE–FcεRI axis—protective surveillance and pro-tumor inflammation—can be spatially juxtaposed and directly compared within a single tissue specimen. This unique feature makes the skin not merely a site of allergic disease but the ultimate model for decoding the context-dependent functions of IgE in tumor immunity. The interdisciplinary field of AllergoOncology, established under the European Academy of Allergy and Clinical Immunology (EAACI) Task Force, has systematically mapped the bidirectional relationship between type 2 immunity and cancer [1,3]. Most work to date, however, has focused on visceral tumors, where tissue accessibility limits the study of spatially resolved IgE effector functions [2,3]. The skin, in contrast, offers a research window that no visceral organ can match: dual exposure to acute and chronic environmental stressors—ultraviolet (UV) radiation, chemical carcinogens, and microbial allergens—and the unique capacity to spatially juxtapose peritumoral surveillance signals with tumor-core inflammatory programs within a single histological section [4,5].

**Figure 1:**
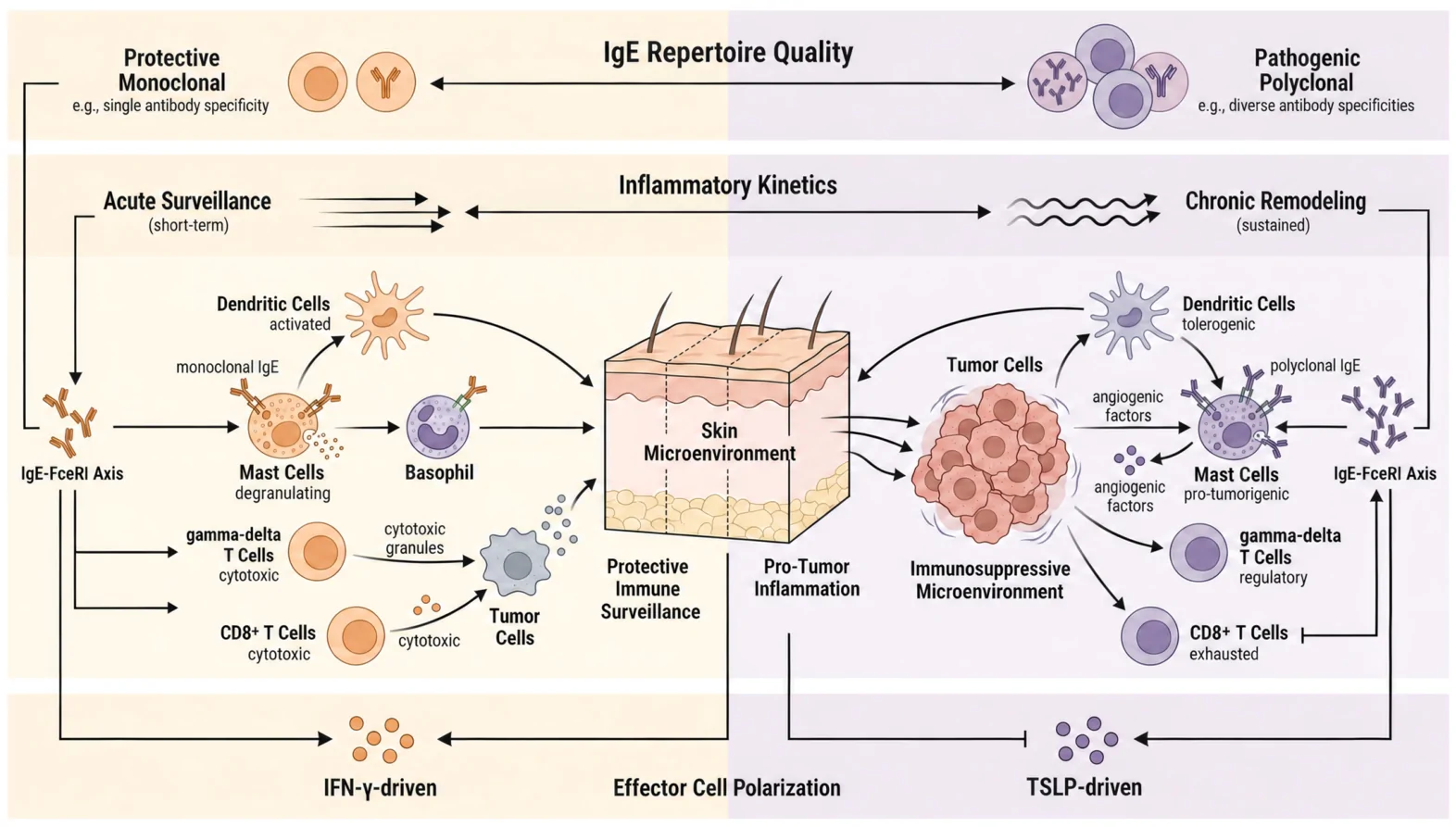
The Skin AllergoOncology conceptual framework. The framework integrates three analytical dimensions—IgE repertoire quality (monoclonal versus polyclonal), inflammatory kinetics (acute versus chronic), and effector-cell polarization state (IFN-γ-driven protective surveillance versus TSLP-driven chronic pro-tumor inflammation)—to determine whether the IgE–FcεRI axis supports protective tumor surveillance or chronic pro-tumor inflammation. The skin is positioned as the only organ in which these two antagonistic programs can be spatially juxtaposed within a single tissue section.

The framework is organized around three analytical dimensions: (1) IgE repertoire quality, distinguishing protective monoclonal from pathogenic polyclonal IgE; (2) inflammatory kinetics, contrasting acute surveillance with chronic remodeling; and (3) effector-cell polarization state, determined by the local cytokine milieu [6,7]. The functional output of the IgE–FcεRI axis—surveillance versus inflammation—emerges from the interplay of these three dimensions. As shown in Figure 1, these three dimensions are integrated into a hierarchical model that positions the skin as the central organ where protective and pathogenic programs are spatially juxtaposed.

References for this Review were identified through searches of PubMed, Embase, and Web of Science using combinations of the search terms ‘IgE’, ‘FcεRI’, ‘mast cell’, ‘basophil’, ‘atopic dermatitis’, ‘skin cancer’, ‘cutaneous malignancy’, ‘melanoma’, ‘squamous cell carcinoma’, ‘basal cell carcinoma’, ‘AllergoOncology’, and ‘tumor immunity’ from January 2000 to April 2026. Additional articles were identified through searches of the authors’ own files and reference lists of key reviews. Only papers published in English were reviewed. Preclinical studies, murine models, human tissue analyses, epidemiological cohort studies, and phase I/II clinical trials were prioritized for inclusion.

We acknowledge the critical missing piece: direct evidence for protective IgE in humans remains absent. However, as we argue throughout this Review, this gap is not a limitation to be lamented but the central scientific question that defines the next decade of the field—a question that is uniquely addressable through the skin model [8,9]. With over 1.5 million new cases of non-melanoma skin cancer (NMSC) annually and rising incidence in UV-exposed regions, the framework’s implications extend from high-income dermatology practices to resource-constrained settings where chronic inflammation management offers a cost-effective cancer prevention strategy [10,11].

## 2. Epidemiological Landscape: From Paradox to Context-Dependence Hypothesis

### 2.1. Atopic Dermatitis and Keratinocyte Carcinoma: Confounding Through the Therapeutic Lens

A meta-analysis of observational studies reports that patients with AD have a 32% increased risk of NMSC, with a pooled standardized rate ratio (SRR) of 1.34 for basal cell carcinoma (BCC) and 1.91 for cutaneous squamous cell carcinoma (cSCC) [12]. These figures have been widely cited as evidence that the AD-associated type 2 immune milieu promotes cutaneous carcinogenesis. However, we argue that this interpretation conflates disease-intrinsic inflammation with treatment-related confounding, a concern that has been raised in recent systematic reviews [13,14].

This association, however, does not in itself constitute evidence that IgE promotes carcinogenesis—a distinction that is essential for the mechanistic hypotheses discussed later. The central confounding challenge is that AD patients with moderate-to-severe disease are routinely exposed to therapies with established or suspected carcinogenic potential: cumulative UV phototherapy doses, long-term topical corticosteroids, calcineurin inhibitors, and, more recently, Janus kinase (JAK) inhibitors [15,16]. A comprehensive systematic review and meta-analysis by Devasenapathy and colleagues demonstrated that topical calcineurin inhibitor use in AD was not significantly associated with increased overall cancer risk, although the confidence intervals were wide and long-term data remained limited [17]. In a population-based case–control study, Cheng and colleagues reported that the association between AD and cSCC was substantially attenuated after adjustment for phototherapy exposure [18]. Comparative studies leveraging psoriasis cohorts, which share intensive phototherapy and immunosuppressive treatment histories, may provide a critical counterfactual [19]. If psoriasis patients receiving comparable therapies exhibit similar NMSC risk elevations, the AD-specific contribution of IgE-mediated inflammation would be called into question. Furthermore, Garritsen and colleagues demonstrated that oral immunosuppressive drug use in AD patients was independently associated with increased NMSC risk, further emphasizing the treatment confounder [20]. A Mendelian randomization study by Liu and colleagues found no causal evidence for an effect of AD on overall cancer risk (OR 1.00, 95% CI 0.94–1.06), further challenging the notion of a direct causal relationship [21].

Collectively, the evidence from treatment-adjusted analyses, comparative cohort studies, and Mendelian randomization does not support a direct causal link between AD-associated IgE elevation and carcinogenesis. Elevated IgE in AD may therefore primarily serve as a biomarker of type 2 inflammation rather than a direct oncogenic driver—a distinction that carries direct implications for the mechanistic models discussed in Section 4.

### 2.2. Allergic History and Melanoma: A Protective Hypothesis Awaiting Proof

A parallel epidemiological literature reports an inverse association between allergic disease history and melanoma risk. Wulaningsih and colleagues found that allergen-specific IgE sensitization, measured prior to cancer diagnosis, was associated with a reduced overall cancer risk (hazard ratio 0.83, 95% CI 0.70–0.99), with a trend toward inverse melanoma risk, particularly in men [22]. These findings, together with earlier ecological studies, have been interpreted as supporting the hypothesis that systemic type 2-biased immune responses may enhance tumor immunosurveillance [23,24]. A comprehensive review by Cui and colleagues further documented site-specific inverse associations between atopy and several cancer types, including melanoma [25]. In a study examining the opposite end of the immunological spectrum, Weller and colleagues reported that adults with undetectable serum IgE levels exhibited a significantly increased hazard of first malignancy, consistent with the hypothesis that a minimal IgE tone contributes to immune surveillance [26]. A systematic review by Muir and colleagues further confirmed that atopic disease was associated with a reduced risk of pancreatic cancer and glioma, though associations with other malignancies remained inconsistent [27].

We caution, however, that the inverse allergy–melanoma association remains an intriguing hypothesis that requires rigorous prospective testing rather than a conclusively established protective relationship. The effect sizes are modest, and most studies lack prospective stratification by allergy subtype, timing of exposure, quantitative IgE levels, and FcεRI^+^ effector cell composition in tumor tissue. The association may reflect shared genetic architecture—for example, loci regulating both atopy susceptibility and immune surveillance—rather than a direct causal pathway [28]. Prospective cohort studies with biospecimen banking are required to move beyond ecological correlation toward mechanistic inference [29].

We propose the following mechanistic hypothesis: if systemic Th2-biased immune responses indeed enhance melanoma immunosurveillance, this protection is likely mediated through the IgE–FcεRI–DC–CD8^+^ T cell axis. Specifically, allergen-specific or autoreactive IgE may engage FcεRI on skin-resident dendritic cells, facilitating antigen capture and cross-presentation of melanoma-associated antigens to CD8^+^ T cells, thereby enhancing tumor-specific cytotoxic responses. This hypothesis predicts that individuals with detectable allergen-specific IgE sensitization would exhibit higher frequencies of melanoma-reactive CD8^+^ T cells in peripheral blood and tumor-infiltrating lymphocytes, and that IgE-mediated cross-presentation efficiency would correlate with improved melanoma-specific survival. These predictions are testable through prospective cohort studies with biospecimen banking and functional immune monitoring.

### 2.3. Serum Total IgE: U-Shaped Curves and Interpretive Pitfalls

The relationship between serum total IgE and cancer risk is not linear. In an EAACI Position Paper, Ferastraoaru and colleagues documented that ultra-low serum IgE levels (below 2.5 kU/L) are associated with increased malignancy risk across multiple cancer types, while moderately elevated IgE may be protective [30]. This U-shaped curve has been interpreted as evidence for a physiological IgE-mediated immunosurveillance function whose absence permits malignant escape [31,32]. The key epidemiological studies examining these associations, with their respective treatment confounder adjustments, are summarized in Table 1.

However, we emphasize that isolated total IgE levels cannot predict individual cancer risk. Serum IgE concentration reflects the aggregate output of systemic allergic sensitization but provides no information about IgE specificity, clonality, or local FcεRI^+^ cellular composition [33]. The same serum IgE level may reflect a protective monoclonal response against tumor-associated antigens or a pathogenic polyclonal response against environmental allergens. This context-dependence forms the central thesis of the next section [34].

## 3. Molecular and Cellular Foundations: Decoding the Dual Outputs of the IgE–FcεRI Axis

### 3.1. IgE and FcεRI Molecular Architecture: Hardware for Context Encoding

The molecular architecture of IgE is uniquely suited to encoding two functionally opposed programs. Recent structural advances have transformed understanding of the IgE–FcεRI interaction, revealing the molecular mechanism of FcεRI activation, the high-resolution architecture of the receptor, and the complete complex structure [35,36,37]. Unlike immunoglobulin G (IgG), IgE adopts a bent conformation in solution, and it binds FcεRI with femtomolar affinity—orders of magnitude higher than the affinity of any IgG isotype for its cognate Fcγ receptors [38]. This high affinity enables IgE to remain tissue-resident for weeks to months, even in the absence of ongoing antigen exposure, providing a molecular basis for sustained immune surveillance [39]. Individual variations in IgE glycosylation patterns regulate its functional properties, contributing to the diversity and complexity of IgE-mediated immune responses [40,41]. In particular, sialylation of IgE has been identified as a critical determinant of allergic pathogenicity, with differentially sialylated IgE glycoforms exhibiting distinct effector functions [41]. A systematic review by McCraw and colleagues further consolidated the evidence that IgE glycosylation—including both N-linked and O-linked glycans—impacts receptor binding, stability, and downstream signaling, underscoring the potential of glycoengineering to modulate IgE function for therapeutic applications [42].

The FcεRI receptor itself exists in two isoforms with distinct signaling capacities. The tetrameric form (αβγ_2_) is expressed on mast cells and basophils and mediates classical allergic effector functions, including degranulation and mediator release. The trimeric form (αγ_2_) is expressed on human dendritic cells (DCs), Langerhans cells, and monocytes, and is primarily associated with antigen uptake and presentation [43,44]. This structural divergence provides the hardware that enables the same receptor system to support both acute immunosurveillance and chronic inflammatory outputs. The β subunit of the tetrameric receptor amplifies signaling through the immunoreceptor tyrosine-based activation motif (ITAM) pathway, a feature absent in the trimeric isoform [43,45].

### 3.2. Skin-Specific Cellular Networks and the Antigen-Focusing Mechanism

**Figure 2:**
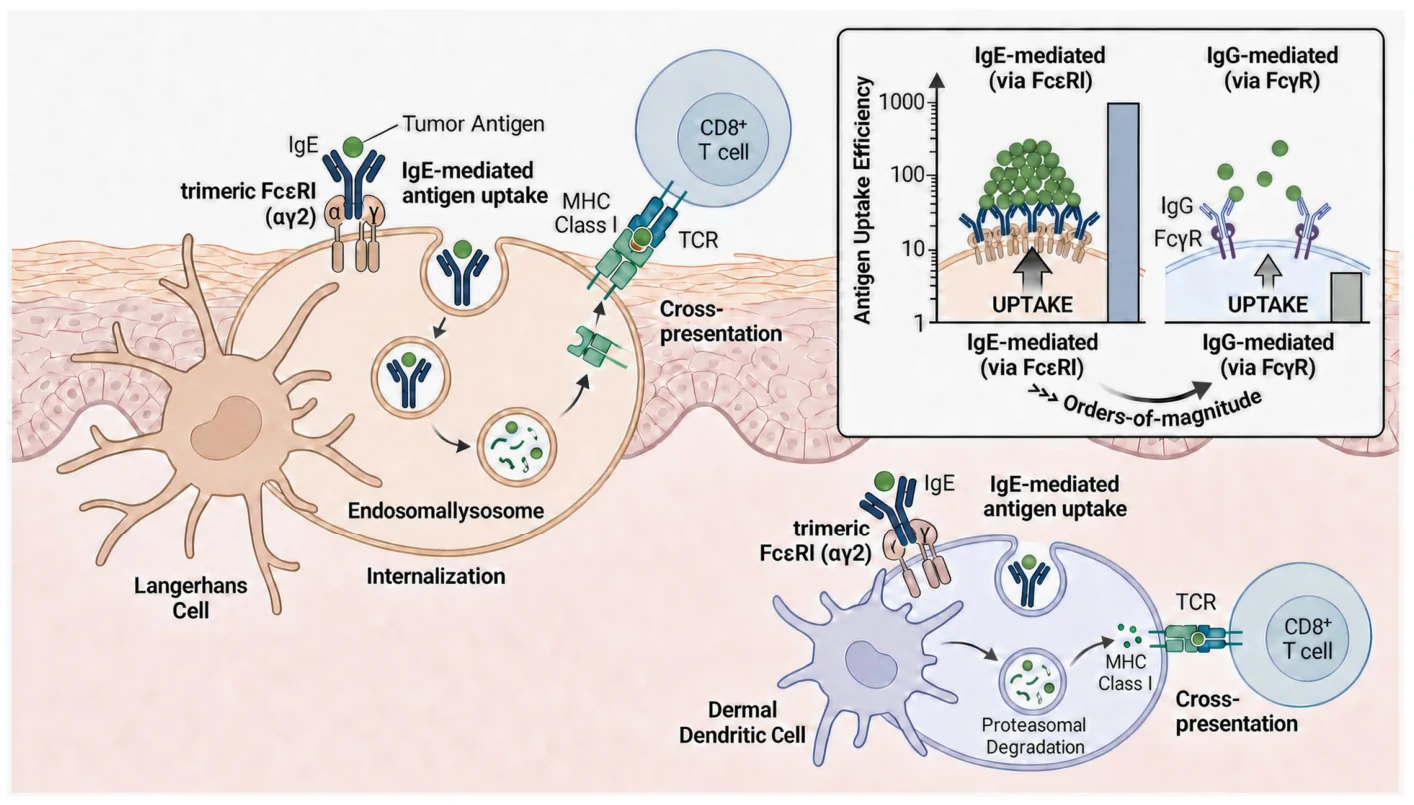
IgE-mediated antigen focusing and cross-presentation by skin dendritic cells. Trimeric FcεRI on Langerhans cells and dermal dendritic cells captures IgE-bound antigens with high avidity. The antigen–IgE–FcεRI complexes are internalized, processed, and cross-presented via major histocompatibility complex class I (MHC-I) molecules to CD8^+^ T cells, enabling the priming of tumor-specific cytotoxic T lymphocytes. This mechanism is orders of magnitude more efficient than IgG-mediated antigen uptake. Abbreviations: TCR, T cell receptor.

Human epidermal Langerhans cells and dermal DCs expressing trimeric FcεRI mediate an “antigen-focusing” effect: by capturing IgE-bound antigens, they concentrate and cross-present peptides to CD8^+^ T cells with an efficiency substantially higher than IgG-mediated uptake [34]. In a seminal study, Platzer and colleagues demonstrated that IgE-mediated cross-presentation by DCs enhances anti-tumor immune responses in vivo, establishing a molecular bridge between the IgE–FcεRI axis and adaptive antitumor immunity [34,46]. Figure 2 illustrates this antigen-focusing mechanism, whereby trimeric FcεRI on Langerhans cells captures IgE-bound tumor antigens and cross-presents them to CD8^+^ T cells.

Critically, the functional output of the same effector cell is not predetermined by its receptor isoform. Mast cells, long considered archetypal mediators of allergic pathology, can release tumor necrosis factor-α (TNF-α) to assist DC priming during acute responses, while in chronic inflammation they predominantly release histamine, which promotes epithelial proliferation [47]. The functional heterogeneity of mast cells is central to understanding their divergent roles in tumor immunity [48,49]. The decisive factor is the microenvironmental cytokine milieu: thymic stromal lymphopoietin (TSLP), abundantly produced by keratinocytes in AD lesions, polarizes mast cells toward a pro-inflammatory, histamine-dominant phenotype [31,50]. TSLP is a key epithelial-derived cytokine upregulated in AD lesional skin in response to barrier disruption, physical injury, or inflammatory stimuli. Upon binding to its receptor (TSLPR), TSLP activates downstream STAT5 and NF-κB pathways, promoting Th2-type immune responses and recruiting mast cells to the skin. TSLP expression is also regulated by epigenetic mechanisms, including promoter demethylation, which contributes to its sustained overexpression in AD. This TSLP-driven milieu creates a feed-forward loop that sustains chronic allergic inflammation and promotes mast cell accumulation and activation. In contrast, interferon-γ (IFN-γ) biases them toward a DC-assisting, TNF-α-dominant output [51].

### 3.3. Generation of Two IgE Lineages: Protective Monoclonal Versus Pathogenic Polyclonal

The two functional programs of the IgE–FcεRI axis are rooted in distinct B-cell differentiation pathways. In a landmark study, Crawford and colleagues showed that epithelial damage induced by topical exposure to the DNA-damaging xenobiotic 7,12-dimethylbenz[a]anthracene triggers a γδ T cell-driven IgE response in which intraepithelial γδ T cells shape the IgE repertoire by supporting specific VDJ rearrangements [32]. The resulting IgE is monoclonal or oligoclonal, autoreactive, and protective against epithelial carcinogenesis. This finding demonstrated that IgE is not merely a mediator of allergic pathology but a component of the epithelial stress surveillance system [8,52].

In contrast, the IgE produced in chronic allergic inflammation is driven by IL-4 and IL-13 from type 2 helper T (Th2) cells and group 2 innate lymphoid cells (ILC2s), generating a polyclonal, low-affinity repertoire directed against environmental allergens [31,53]. These two IgE lineages differ fundamentally in their origin, specificity, and function. The protective lineage is characterized by γδ T cell-dependent generation in response to acute epithelial damage and DNA-damaging xenobiotics, monoclonal or oligoclonal VDJ rearrangements with unique CDRH3 characteristics, autoreactivity against stress-induced or tumor-associated self-antigens, and high-affinity FcεRI binding that facilitates cross-presentation by dendritic cells and CD8^+^ T cell priming. The pathogenic lineage, in contrast, is characterized by IL-4/IL-13-dependent generation in chronic Th2-type inflammation, a polyclonal low-affinity repertoire directed against environmental allergens, predominant engagement of mast cells and basophils, and promotion of histamine release and epithelial proliferation.

The divergent VDJ repertoires and antigen affinities of these two lineages determine whether the IgE–FcεRI axis supports immunosurveillance or chronic inflammation—a distinction that is not captured by measuring total serum IgE [34]. The tissue microenvironment thus acts as a critical determinant of IgE repertoire quality.

## 4. Context-Dependent Dual Roles: From Mechanism to Human Inference

### 4.1. Acute Stress Surveillance: Proven in Mice, Testable in Humans

The murine protective pathway now has a well-characterized causal chain: epithelial damage induced by topical carcinogens releases damage-associated molecular patterns (DAMPs) that act as “danger signals,” triggering γδ T cells in the intraepithelial compartment, which in turn drive class switching to IgE in local B cells [32,54]. The resulting autoreactive IgE, via FcεRI, engages DCs for antigen cross-presentation, which in turn primes CD8^+^ T cells for antitumor effector function [34]. Genetic experiments by Nigro and colleagues confirmed that IgE ablation rendered mice more susceptible to transplantable tumor growth, while FcεRI knockout abrogated most of the IgE-mediated protection, and CD8^+^ T cell depletion similarly abolished the protective effect [55]. Recent work by Ding and colleagues demonstrated that long-lived IgE plasma cells persist in secondary lymphoid tissues using a navitoclax-sensitive survival program, providing mechanistic insight into the durability of IgE-mediated surveillance [56].

The key question is whether a homologous protective program operates in human skin. Indirect evidence is suggestive: in human cSCC, high expression of the gene encoding FcεRIα correlates with favorable disease prognosis [32]. More recently, Matin-Dokht and colleagues reported that FcεRI^+^ tumor-infiltrating leucocytes, including conventional DC type 2 cells and plasmacytoid DCs, are enriched in human cSCC tissue [57]. However, these data remain correlative in humans; direct functional evidence for protective IgE in human skin cancer is absent [58].

The absence of such evidence is not a limitation to be lamented but the central scientific question that defines the next decade of Skin AllergoOncology—a question uniquely addressable through the skin model. We propose a testable hypothesis: if a functional IgE–FcεRI–CD8^+^ T cell immune synapse exists in human cSCC, then spatial transcriptomics should detect statistically significant co-localization of IgE^+^ B cells, FcεRI^+^ DCs, and clonally expanded CD8^+^ T cells in good-prognosis tumors. This design transforms static histological evidence into dynamic functional inference [59]. Spatial transcriptomics now enables genome-wide expression profiling with spatial resolution, making this hypothesis directly testable [60,61]. Recent studies by Gim and colleagues and Prakrithi and colleagues have demonstrated the power of these approaches to resolve spatially organized immune cellular ecosystems in cutaneous malignancies [62,63].

### 4.2. Chronic Inflammation-Driven Carcinogenesis: Closing the Human Evidence Loop

The chronic inflammation → polyclonal IgE → mast cell/basophil → histamine → epithelial proliferation chain is supported by convergent evidence from AD pathogenesis. AD lesional skin is characterized by TSLP overexpression, mast cell hyperplasia, and elevated histamine levels [45,48]. Histamine has well-documented pro-proliferative effects on keratinocytes via H_1_ and H_2_ receptors, and chronic mast cell degranulation contributes to the epidermal hyperplasia that characterizes chronic eczematous lesions [47,64].

Importantly, the mechanistic evidence implicating IgE in chronic inflammation-driven carcinogenesis must be interpreted alongside the epidemiological confounders discussed in Section 2.1: it remains unclear whether the increased NMSC risk in AD reflects IgE-mediated pathology, the cumulative effects of immunosuppressive therapies, or both [13,20,65]. This ambiguity can only be resolved through experimental models that genetically or pharmacologically isolate IgE from the broader type 2 inflammatory milieu. AD is a complex disease in which IgE elevation coexists with barrier dysfunction due to filaggrin mutations, microbial dysbiosis, and a network of cytokines—IL-4, IL-13, IL-31, and TSLP—that independently promote keratinocyte proliferation [66,67]. We argue that the field must move beyond the “epiphenomenon versus direct culprit” debate by employing IgE-knockout or anti-IgE-treated mouse models of AD-associated carcinogenesis. Indeed, genetic ablation of IgE in mice has been shown to increase susceptibility to transplantable tumor growth, while reconstitution with IgE restored protection, establishing a direct causal role for IgE in tumor immunosurveillance [31,32].

### 4.3. Unified Theoretical Model: The Skin Microenvironment as a Functional Program Switch

**Figure 3:**
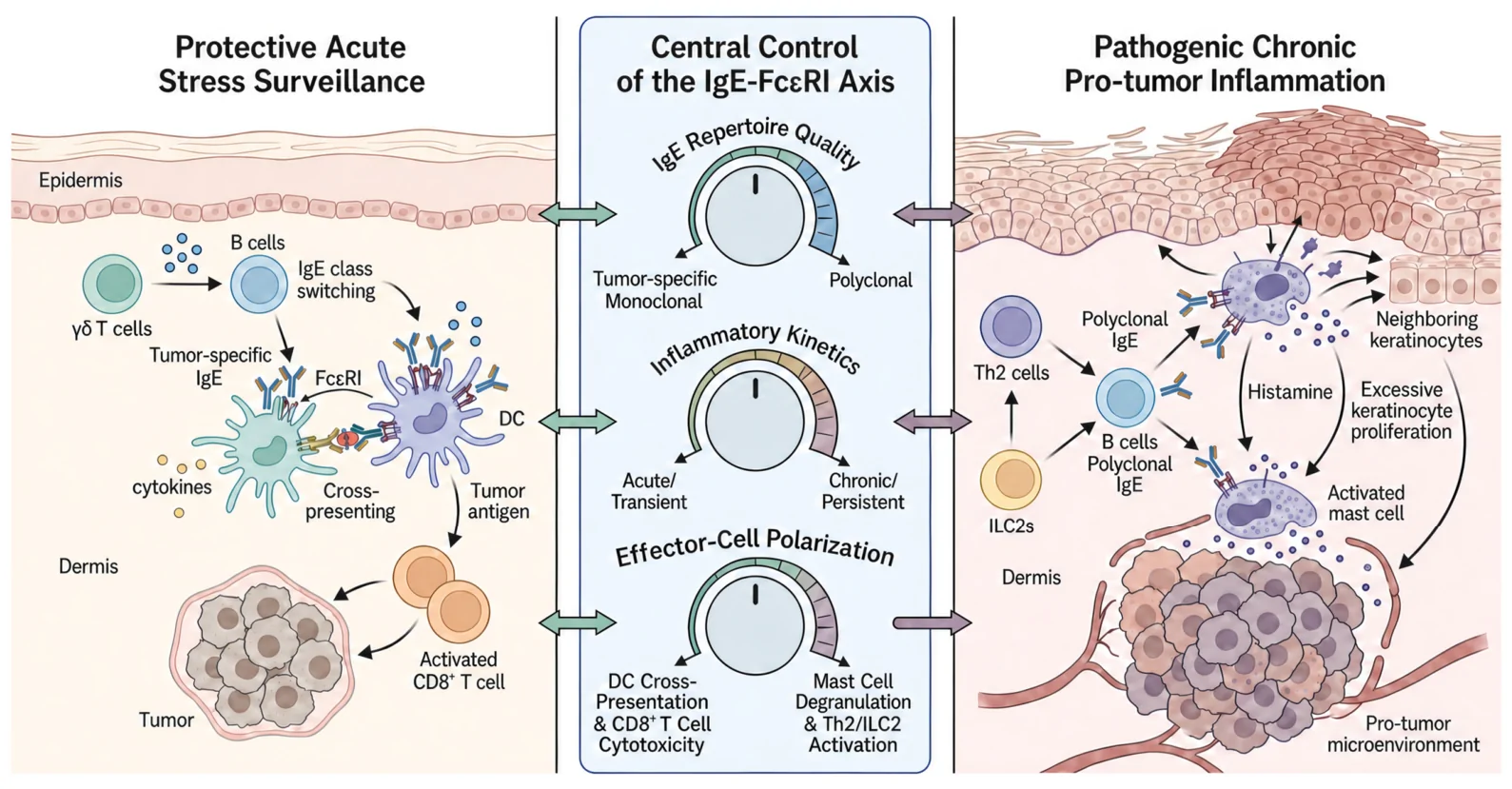
Contrasting functional programs of the IgE–FcεRI axis in the skin. The upper panel depicts the acute stress surveillance program: epithelial damage → γδ T cell-driven monoclonal IgE → FcεRI^+^ DC cross-presentation → CD8^+^ T cell antitumor response. The lower panel depicts the chronic pro-tumor inflammation program: Th2-driven polyclonal IgE → mast cell/basophil activation → histamine release → epithelial proliferation. The three microenvironmental dimensions determining which program prevails are highlighted: IgE repertoire quality, inflammatory kinetics, and effector-cell polarization.

Synthesizing the evidence, we propose a unified model in which the skin microenvironment functions as a three-dimensional program switch governing IgE–FcεRI output [68]. The three decisive dimensions are: (1) IgE repertoire quality—monoclonal, autoreactive IgE initiates surveillance while polyclonal, allergen-specific IgE drives inflammation; (2) inflammatory kinetics—acute, transient signals favor DC-mediated priming and CD8^+^ T cell activation, whereas chronic, sustained signals favor mast cell-mediated tissue remodeling and epithelial proliferation; and (3) effector-cell polarization—the local cytokine milieu (TSLP versus IFN-γ) determines whether the same mast cell population releases histamine or TNF-α [69]. The skin uniquely enables spatial juxtaposition of both programs. Figure 3 provides a side-by-side comparison of these two contrasting functional programs.

## 5. Clinical Translation Frontiers: Harnessing the Therapeutic Potential and Managing Risks of the IgE Axis

### 5.1. Risk-Stratification Biomarker Panels

The Skin AllergoOncology framework provides the conceptual basis for a potentially clinically applicable decision tool that integrates isolated biomarkers into a coherent risk-stratification algorithm [70]. We propose a testable risk-stratification algorithm: low serum total IgE combined with low tumor tissue FcεRIα expression defines a high-risk profile for skin cancer; conversely, high serum IgE combined with high FcεRIα expression and a protective repertoire signature defines a low-risk profile. This algorithm integrates systemic (serum IgE), tissue (FcεRIα expression), and molecular (repertoire signature) evidence tiers, and is amenable to prospective validation in existing skin cancer cohorts [71]. Table 2 summarizes the current landscape of engineered IgE antibodies in clinical and preclinical development.

### 5.2. Engineered IgE Antibodies: Urticaria as a Pharmacodynamic Biomarker

Engineered IgE antibodies represent the most direct translational application of the Skin AllergoOncology concept. As reviewed by Brewer and colleagues, the strategic re-routing of IgE for cancer therapy has emerged as a promising frontier, with IgE antibodies offering distinct advantages over IgG-based therapeutics through their engagement of a broader effector cell network [77]. Unlike IgG-based therapeutics, IgE antibodies engage a distinct effector cell network—mast cells, basophils, DCs, and eosinophils—that is enriched in the skin and other barrier tissues [72,78]. The first-in-class IgE therapeutic, MOv18 IgE, targeting folate receptor α, has completed phase I clinical evaluation and demonstrated a favorable safety profile with no evidence of anaphylaxis [72]. Recent biophysical characterization has confirmed the monomeric purity and stability of MOv18 IgE, supporting its manufacturability and clinical development [78]. In December 2024, Epsilogen announced the initiation of a Phase Ib trial (NCT06547840) evaluating MOv18 IgE in patients with platinum-resistant ovarian cancer, enrolling 45 patients in a dose escalation and expansion design [79]. CSPG4-IgE, targeting chondroitin sulfate proteoglycan 4 on melanoma cells, demonstrated pro-inflammatory anti-cancer effects in preclinical models, including enhanced macrophage infiltration and enriched pro-inflammatory signaling pathways in the tumor microenvironment [73,74,80].

Because therapeutic IgE engages FcεRI on skin-resident mast cells, the resulting mast cell degranulation manifests clinically as transient urticaria—a visible, non-invasive pharmacodynamic readout of successful local FcεRI network engagement. Treatment-emergent urticaria should therefore be reframed as a quantifiable biomarker of target engagement [81]. The severity and timing of this reaction correlate with the extent of FcεRI occupancy on skin-resident mast cells, providing a quantifiable, patient-specific readout for dose titration [82]. Urticaria provides a real-time window for precision dose escalation that no other immunoglobulin class can offer, transforming a perceived toxicity into a clinical asset [83].

Beyond passive administration, a conceptually complementary strategy has emerged that leverages the IgE–FcεRI axis for cell-based drug delivery. Xu and colleagues demonstrated that mast cells sensitized with tumor-specific IgE can be harnessed as living drug carriers, delivering therapeutic payloads precisely to the tumor microenvironment while simultaneously augmenting local anti-tumor immune responses [66]. This approach exploits the same FcεRI-dependent targeting mechanism but repurposes it for active cellular delivery—a strategy that may synergize with systemic IgE antibody therapy, particularly in the context of cutaneous malignancies where mast cells are abundantly resident.

The accessibility of cutaneous malignancies further enables skin-specific delivery technologies, including hydrogel sustained-release systems and microneedle patches, to overcome the tumor stroma penetration barriers that limit large-molecule antibody therapeutics [84,85]. The principles of transdermal immunomodulation—leveraging the skin’s rich immune network for localized therapeutic delivery—provide a conceptual foundation for these approaches [86]. More broadly, the rational design of biomaterials to control immune signaling within the skin—by modulating the spatiotemporal presentation of immunomodulatory cues—offers a powerful framework for enhancing the efficacy of locally delivered IgE-based therapies [70]. This biomaterial-directed approach aligns with the Skin AllergoOncology concept that the tissue microenvironment, rather than the molecular entity alone, governs therapeutic outcome.

### 5.3. The Shadow of Existing Therapies: Long-Term Oncological Risks of Anti-Allergic Biologics

The expanding indications for anti-allergic biologics—omalizumab (anti-IgE) and dupilumab (anti-IL-4Rα)—in both pediatric and adult populations raise an urgent, unresolved clinical question: does long-term pharmacological suppression of the IgE–FcεRI axis increase cancer risk? Current pharmacovigilance data provide no definitive evidence of increased malignancy, but the absence of evidence does not constitute evidence of safety, particularly given the decades-long latency of solid tumors [83,87]. The Skin AllergoOncology framework predicts that suppression of the protective IgE–FcεRI surveillance program could, over time, impair the immunological detection and elimination of nascent malignant clones [88]. For omalizumab, pooled analyses and real-world registry data have consistently reported no association with increased cancer risk [80]. For dupilumab, although most retrospective studies report no overall increase in cancer risk, concerns remain regarding a possible association with cutaneous T-cell lymphoma, highlighting the need for prospective monitoring [89]. We call for the establishment of a global pharmacovigilance system with mandatory long-term follow-up registries to prospectively monitor cancer incidence in patients receiving anti-IgE and anti-type 2 biologics.

This is not merely a research recommendation but a clinical imperative. The global cancer burden disproportionately affects low- and middle-income countries, where access to advanced immunotherapies remains severely limited [90]. In low-resource settings where biologics are inaccessible, the framework’s implications are equally critical: effective control of chronic allergic inflammation through affordable topical therapies may represent the most feasible path to reducing inflammation-driven skin cancer risk [91].

**Figure 4:**
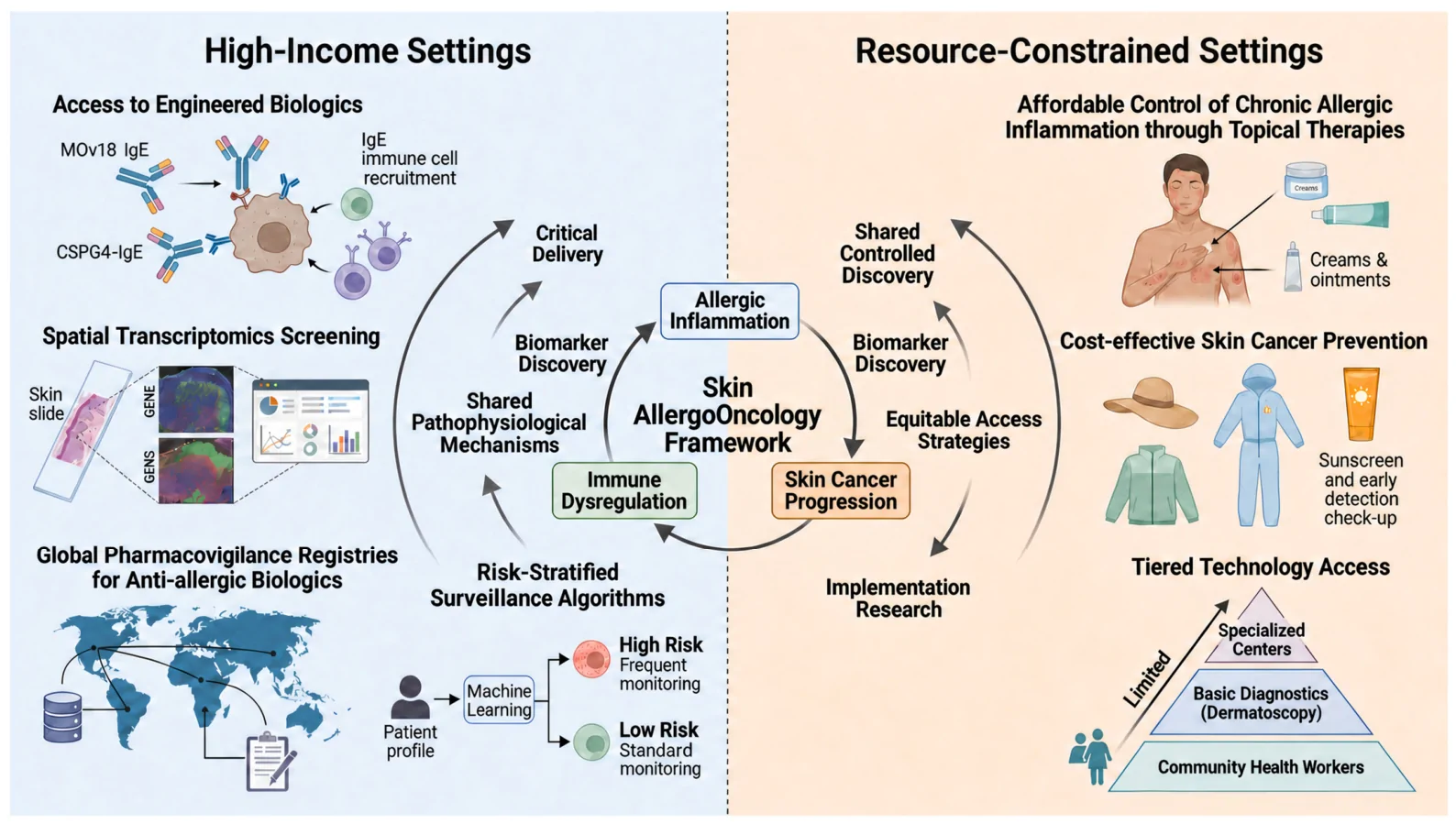
Global health spectrum of Skin AllergoOncology translational implications. The left panel depicts high-resource settings where engineered IgE antibodies, spatial transcriptomics-based risk stratification, and biologics safety registries are feasible. The right panel depicts low-resource settings where affordable control of chronic inflammation represents the primary cancer prevention strategy.

Figure 4 illustrates the global health spectrum of these translational implications, contrasting high-resource settings where engineered IgE antibodies and spatial transcriptomics are accessible with low-resource settings where affordable inflammation control remains the primary prevention strategy.

## 6. Core Challenges and Future Roadmap: Identifying the Protective IgE Signature

### 6.1. The Single Bottleneck: Can a Human Protective IgE Signature Be Identified?

The identification of a human “protective IgE signature” is the fundamental prerequisite for all clinical translation in Skin AllergoOncology [92]. Without this signature, protective IgE remains a murine phenomenon, epidemiological associations remain unactionable, and engineered IgE therapies lack a companion diagnostic for patient selection [93]. The signature is defined by two components: an antigen spectrum, encompassing the tumor-associated or stress-associated antigens recognized by protective IgE, and an Fc-glycosylation pattern, such as differential sialylation or fucosylation that modulates FcεRI binding affinity and effector function [40,41].

Several lines of evidence support the use of Fc glycosylation as a defining parameter of the protective IgE signature. In IgG biology, Fc glycosylation, particularly sialylation and fucosylation, critically determines antibody effector function by modulating Fcγ receptor binding and complement activation. A parallel mechanism is increasingly recognized for IgE, where glycan composition directly influences FcεRI binding affinity and downstream signaling. Shade and colleagues demonstrated that IgE sialylation is a determinant of allergic pathogenicity, with differentially sialylated IgE glycoforms exhibiting distinct effector functions, establishing glycosylation as a functional feature rather than a merely structural one [41]. Importantly, glycosylation patterns are quantitatively measurable using established mass spectrometry-based platforms, rendering them clinically tractable as biomarkers. Kumari and colleagues further showed that N-linked glycosylation at position N275 of the IgE heavy chain is essential for IgE stability and secretion, and that deglycosylated IgE variants exhibit superior efficacy in vitro [94]. Collectively, these findings suggest that the Fc-glycosylation pattern of IgE may serve as a tunable parameter that distinguishes protective from pathogenic IgE, and that glycan modifications are not only biomarkers but also potential therapeutic targets.

### 6.2. The Five-Year Dual-Engine Roadmap

The identification of a human protective IgE signature requires a coordinated, multi-phase effort. We propose a five-year dual-engine roadmap that combines tissue-based screening with in situ antigen capture, with the two engines operating sequentially and complementarily.

#### 6.2.1. Engine One: Reverse-Translational Screening (Years 1–2)

The first engine leverages existing skin cancer cohorts to transform biomarker discovery from blind search into guided prospecting. Using spatial transcriptomics, patients with active IgE–FcεRI–CD8^+^ T cell immune synapse signals can be pre-screened from large cSCC cohorts [60,62]. Paired serum samples from “synapse-positive” patients are then retrospectively analyzed for IgE antigen repertoires using antigen microarrays or phage display, and for Fc-glycosylation patterns via mass spectrometry [94]. This reverse-translational approach—tissue → serum—narrows the search space from the entire IgE repertoire to a candidate set of tumor-associated specificities [95].

#### 6.2.2. Engine Two: In Situ Skin-Patch Antigen Capture (Years 3–5)

The second engine exploits the unique accessibility of the skin to develop a non-invasive antigen capture system. FcεRIα protein-containing patches, applied to premalignant lesions such as actinic keratoses or early cSCC, capture locally bound IgE–antigen complexes in situ [96]. Subsequent mass spectrometry identifies the captured antigens, bypassing the complex B-cell cloning and recombinant expression steps that have historically bottlenecked IgE repertoire analysis [97]. This approach enables true in vivo protective IgE antigen spectrum discovery directly from human skin. The conceptual foundation for this approach is the antigen-focusing mechanism of FcεRI itself [34]: the same high-affinity binding that enables DCs to capture IgE–antigen complexes for cross-presentation can be exploited for diagnostic antigen capture.

### 6.3. Integration and the Five-Year Goal

**Figure 5:**
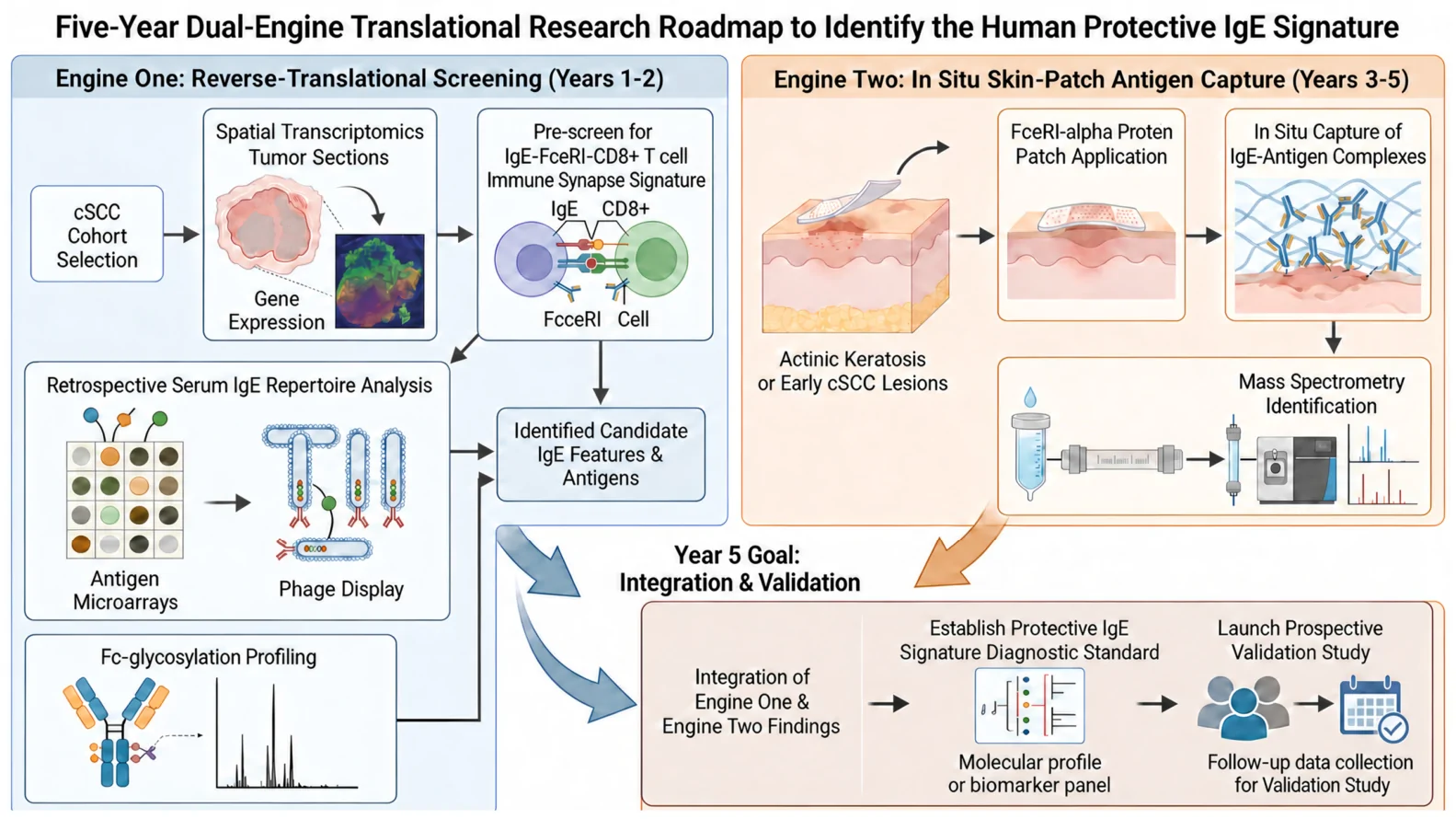
Five-year dual-engine roadmap for protective IgE signature identification. Engine One (Years 1–2): reverse-translational screening using spatial transcriptomics on existing cSCC cohorts to identify IgE–FcεRI–CD8^+^ T cell immune synapses, followed by retrospective serum IgE repertoire and glycosylation analysis. Engine Two (Years 3–5): in situ skin-patch antigen capture using FcεRIα-containing patches applied to premalignant lesions, followed by mass spectrometry identification. Year 5: integration to establish a protective IgE signature diagnostic standard and launch prospective validation.

The ultimate Year 5 deliverable is the integration of Engine One and Engine Two discoveries to establish a “protective IgE signature” diagnostic standard, defined by a specific antigen spectrum and a specific Fc modification, and the launch of prospective validation in an independent cohort [98]. The broader implications extend beyond skin cancer: the fundamental functional principles of the IgE–FcεRI axis revealed by the skin model are expected to apply to all barrier-organ tumors rich in FcεRI^+^ effector cells, from the lung to the gastrointestinal tract [99]. Figure 5 provides a visual overview of the five-year dual-engine roadmap.

## 7. Limitations

Several limitations of this review warrant consideration. First, direct functional evidence for a protective role of IgE in humans is currently lacking, as most insights come from murine models and human correlative studies. Second, the epidemiological link between AD and keratinocyte carcinoma is based largely on observational data, and the potential confounding influence of immunosuppressive treatments—including phototherapy, topical corticosteroids, calcineurin inhibitors, and JAK inhibitors—cannot be completely ruled out. Third, although we outline a five-year dual-engine roadmap aimed at identifying the human protective IgE signature, the technologies involved, such as spatial transcriptomics and in situ skin-patch antigen capture, remain under development and will require prospective validation in independent patient cohorts. Fourth, the available evidence on IgE–FcεRI biology in cutaneous malignancies comes predominantly from European and North American populations, limiting the generalizability of findings to other ethnicities and geographic regions.

## 8. Conclusions

This Review makes three distinct contributions to the field. First, it proposes the novel ‘Skin AllergoOncology’ conceptual framework, which provides a unified theoretical model to resolve the long-standing epidemiological paradox linking allergic inflammation and cutaneous malignancy. Second, it defines the skin as the only human organ in which two antagonistic IgE–FcεRI programs can be spatially juxtaposed and directly compared within a single tissue specimen, an approach that establishes the skin as the ultimate model for decoding context-dependent IgE functions. Third, it outlines a five-year dual-engine translational roadmap combining reverse-translational spatial transcriptomics with in situ skin-patch antigen capture to identify the human protective IgE signature, thereby translating mechanistic insight into a testable clinical strategy.

The Skin AllergoOncology framework reveals that the functional outcome of the IgE axis is not predetermined by its molecular hardware but is dynamically programmed by the tissue microenvironment. This framework offers a coherent mechanistic model that reconciles conflicting epidemiological observations, and while it has limitations, the absence of a validated human protective IgE signature should be viewed not as a deficit but as an opportunity for future investigation. The Skin AllergoOncology framework, through its unique spatial and mechanistic vantage point, is well positioned to address this challenge.

## Figures and Tables

**Table 1:** Key epidemiological studies of atopic dermatitis and skin cancer risk, stratified by treatment confounder adjustment.

Study	Cancer Type	Risk Estimate	Treatment Adjustment	Key Confounder Noted
Gandini et al. (2016) [12]	BCC	SRR 1.34	No	Atopic disease severity
Gandini et al. (2016) [12]	cSCC	SRR 1.91	No	Phototherapy exposure
Cheng et al. (2015) [18]	cSCC	Attenuated	Yes	Phototherapy, topical steroids
Garritsen et al. (2017) [20]	NMSC	Elevated	Yes	Oral immunosuppressants
Wulaningsih et al. (2016) [22]	Melanoma	HR 0.83	No	IgE sensitization status
Ferastraoaru et al. (2020) [30]	Pan-cancer	U-shaped	No	Ultra-low IgE threshold
Liu et al. (2023) [21]	Pan-cancer	OR 1.00	MR design	Genetic IVs

NMSC, non-melanoma skin cancer; BCC, basal cell carcinoma; cSCC, cutaneous squamous cell carcinoma; SRR, standardized rate ratio; OR, odds ratio; HR, hazard ratio; MR, Mendelian randomization; IVs, instrumental variables.

**Table 2:** Engineered IgE antibodies in clinical and preclinical development for solid tumors.

Antibody	Target Antigen	Indication	Stage	Key Feature
MOv18 IgE	Folate receptor α	Ovarian carcinoma	Phase I/Ib	First-in-class IgE therapeutic [72,73] *
CSPG4-IgE	CSPG4	Melanoma	Preclinical	Pro-inflammatory TME remodeling [74]
Trastuzumab-IgE	HER2/neu	Breast carcinoma	Preclinical	ADCC via eosinophils [75]
Anti-CD38 IgE	CD38	Multiple myeloma	Preclinical	Fully human IgE construct [76]

* [72] Shen et al. (Phase I); [73] Park & Lin (Phase Ib). TME, tumor microenvironment.

## Data Availability

No new data were created or analyzed in this study. Data sharing is not applicable to this article.

## Abbreviations

The following abbreviations are used in this manuscript:

IgE	Immunoglobulin E
FcεRI	Fc fragment of immunoglobulin E, high affinity receptor I
IgG	Immunoglobulin G
FcγR	Receptors for the Fc fragment of immunoglobulin G
AD	Atopic dermatitis
EAACI	The European Academy of Allergy and Clinical Immunology
UV	Ultraviolet
TSLP	Thymic stromal lymphopoietin
IFN-γ	Interferon-gamma
NMSC	Non-melanoma skin cancer
BCC	Basal cell carcinoma
cSCC	Cutaneous squamous cell carcinoma
SRR	Standardized rate ratio
OR	Odds ratio
HR	Hazard ratio
MR	Mendelian randomization
IVs	Instrumental variables
DCs	Dendritic cells
ILC2s	Group 2 innate lymphoid cells
Th1/2	Type 1/Type 2 helper T cell
TME	Tumor microenvironment
ITAM	Immunoreceptor tyrosine-based activation motif
MHC-I	Major histocompatibility complex class I
TCR	T cell receptor
IL	Interleukin
JAK	Janus kinase
TNF-α	Tumor necrosis factor-α
